# ﻿A new species of *Entedon* Dalman (Hymenoptera, Eulophidae) and three newly recorded species from China

**DOI:** 10.3897/zookeys.1172.104676

**Published:** 2023-07-21

**Authors:** Ming-Rui Li, Cheng-De Li

**Affiliations:** 1 Jilin Provincial Key Laboratory of Insect Biodiversity and Ecosystem Function of Changbai Mountains, Beihua University, Jilin, 132013, China Beihua University Jilin China; 2 School of Forestry, Northeast Forestry University, Harbin, 150040, China Northeast Forestry University Harbin China

**Keywords:** Chalcidoidea, Entedoninae, natural enemy, new species records, parasitoid wasp, taxonomy

## Abstract

In this paper, a new species of *Entedon* Dalman, *E.flavifemur***sp. nov.** is described from Tibet and three species, *E.albifemur* Kamijo, *E.crassiscapus* Erdös, and *E.nomizonis* Kamijo are reported from China for the first time. A detailed description and illustrations of the new species are provided, as well as diagnoses and illustrations of the three newly recorded species.

## ﻿Introduction

Species of genus *Entedon* Dalman, 1820 (Hymenoptera, Eulophidae, Entedoninae) are considered to primarily parasitize the larvae of weevils, bark beetles (Coleoptera, Curculionidae), and bean weevils (Coleoptera, Chrysomelidae, Bruchinae) which bore into tree trunks, wood, and seeds (Schauff 1988; Askew 1991). The genus *Entedon* can be separated from other genera in Entedoninae by the following characters: head and thorax strongly sclerotized, with conspicuous reticulation; antenna usually with 3-segmented funiculus and 2-segmented clava, and sometimes with five flagellar segments separated from each other, not forming an obvious clava; frontal sulcus often absent, although well developed in some species; mandibles bidentate; scutellum convex; propodeum mostly smooth (rarely reticulate) with single median carina that may split and diverge anteriorly; propodeal spiracle on an elevated area; and fore wing with cubital setal line and basal setal line usually absent or incomplete, rarely complete (Gumovsky 1997; Majeed et al. 2021).

The genus *Entedon* contains 187 species worldwide, of which 185 species were recorded in the Universal Chalcidoidea Database (Noyes 2019), and two species were described more recently by Majeed et al. (2021) from India. There are 14 species known from China: seven species (*E.betulae* Yang, *E.broussonetiae* Yang, *E.pini* Yang, *E.pumilae* Yang, *E.tumiditempli* Yang, *E.wilsonii* Yang, *E.yichunicus* Yang) were described by Yang (1996) during an investigation of parasitic wasps on bark beetles in China; two species (*E.epicharis* Huang, *E.zanara* Walker) were reported by Wang et al. (1991), both reared from the larvae of *Agrilussuvorovi* Obenberger; five species (*E.abdera* Walker, *E.gracilior* Graham, *E.methion* Walker, *E.punctiscapus* Thomson, *E.squamosus* Thomson) were reported by Zhu and Huang (2001), Zhu and Huang (2002), and Zhang et al. (2007) during the taxonomic study on Eulophidae from Zhejiang, Guangxi, and Gansu provinces of China, respectively.

In this paper we record four additional species: *E.flavifemur* is described as new to science; and *E.albifemur* Kamijo, 1988, *E.crassiscapus* Erdös, 1944 and *E.nomizonis* Kamijo, 1988 are reported from China for the first time. The detailed description and illustrations of the new species, diagnoses and illustrations of three new recorded species are provided.

## ﻿Materials and methods

Specimens were collected by sweep nets, yellow-pan traps, and Malaise traps from northeast and southwest China, and were fixed on triangular cards or dissected and mounted in Canada Balsam on slides following methods described by Noyes (1982). Photographs were taken with an Aosvi AO-HK830-5870T digital microscope or a digital CCD camera attached to an Olympus BX51 compound microscope. The quality of these images was improved by using Helicon Focus 7 and Adobe Photoshop 2020. Measurements were made using the built-in software of Aosvi AO-HK830-5870T.

Terminology follows the Hymenoptera Anatomy Consortium (2023), and the following abbreviations are used:

**C1–2** clavomeres 1–2;

F1–3 funiculars 1–3;

**HE** height of eye;

**MS** malar space;

**MV** marginal vein;

**OOL** minimum distance between a posterior ocellus and corresponding eye margin;

**PMV** postmarginal vein;

**POL** minimum distance between posterior ocelli;

SMV submarginal vein;

**STV** stigmal vein;

**WM** width of mouth opening.

Type material is deposited in the insect collections at Northeast Forestry University (**NEFU**), Harbin, China. Abbreviations for other depositories:

**EIHU**Entomological Laboratory, Hokkaido University, Japan;

**HNHM**Hungarian Natural History Museum, Budapest, Hungary.

## ﻿Taxonomic accounts

### ﻿ Entedon flavifemur

Taxon classificationAnimaliaHymenopteraEulophidae

Li & Li sp. nov.

D146022B-0161-546E-B934-231CB59529A9

https://zoobank.org/B5C82D77-CB83-4C07-96D4-ED165CD8165C

Figs 1 , 2A–K

#### Type material.

***Holotype***: ♀ [NEFU; on card], China, Tibet, Lhasa City, Linzhou County, 30–31.V.2015, leg. Ye Chen and Chao Zhang, by yellow-pan trapping. ***Paratypes***: 4♀: 1♀ [NEFU; on slide], China, Tibet, Medog County, Damu Village, 15–18.V.2017, leg. Zhaxi, by Malaise trapping; 2♀ [NEFU; on cards], China, Tibet, Medog County, Damu Village, 25.V.–1.VI.2017, leg. Zhaxi, by Malaise trapping; 1♀ [NEFU; on card], CHINA, Tibet, Medog County, Damu Village, 15–22.VI.2017, leg. Zhaxi, by Malaise trapping.

#### Diagnosis.

**Figure 1.**
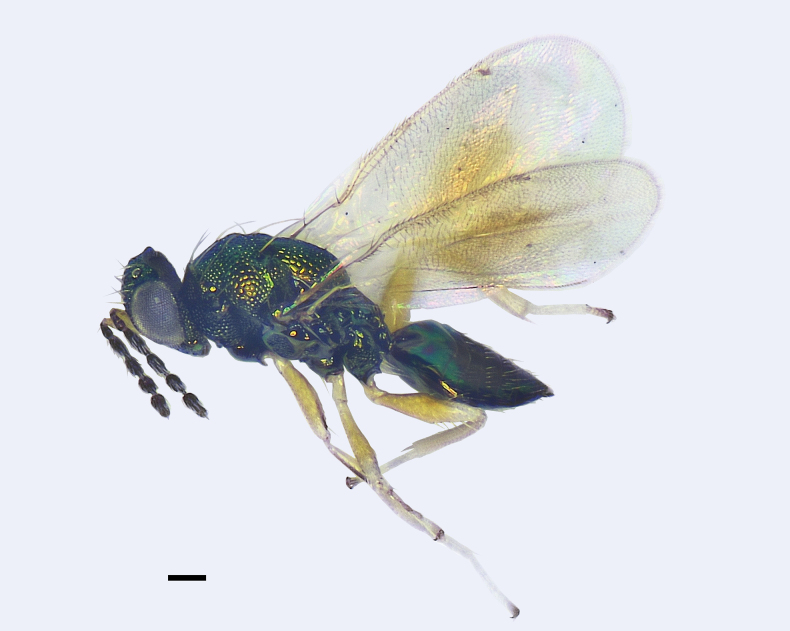
*Entedonflavifemur* Li & Li, sp. nov., holotype, female, habitus in lateral view. Scale bar: 200 μm.

**Figure 2.**
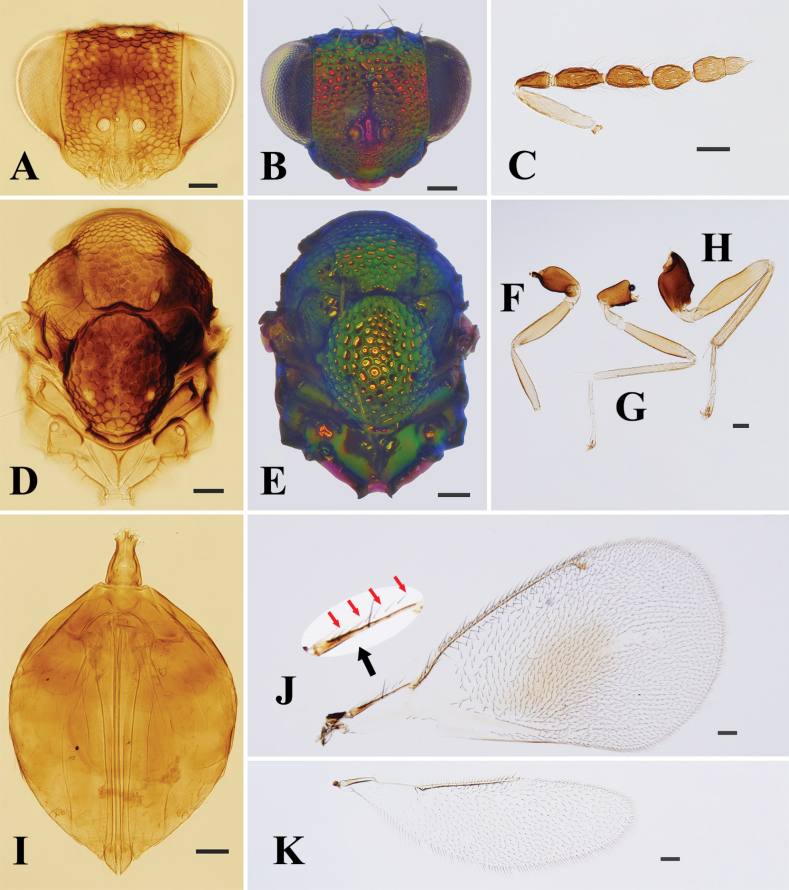
*Entedonflavifemur* Li & Li, sp. nov., paratype, female, on slide **A, B** head, frontal view **C** antenna **D, E** mesosoma **F–H** fore, mid and hind leg, respectively **I** metasoma **J** fore wing, red arrows show the row of short setae on lower surface **K** hind wing. Scale bars: 100 μm.

**Female.** Scape and all femora and tibiae yellow to pale yellow (Figs 1, 2C, F–H); propodeum smooth, without reticulation (Fig. 2D, E); fore wing with an oval-shaped infuscate spot below MV (Figs 1, 2J); lower surface of costal cell with a row of short setae close to SMV and extending from base to 2/3 length of the cell (Fig. 2J).

#### Description.

**Female.** Body length 2.2–2.4 mm, mainly metallic green. Vertex and face metallic green, the latter with golden-red reflections, and interantennal area with golden-violet reflections (Fig. 2B). Eyes and ocelli dark reddish brown. Antenna with scape yellow to pale yellow, pedicel and flagellum dark brown with weak bluish green reflections (Figs 1, 2C). Mesosoma and metasoma metallic green, with golden-yellow or copper reflections (Fig. 2E). All legs with coxae metallic green; trochanters, femora, and tibiae yellow to pale yellow; first three tarsomeres white to pale yellow and 4^th^ tarsomere brown to dark brown (Figs 1, 2F–H). Fore wing with an oval-shaped infuscate spot below MV (Fig. 2J).

***Head*** (Fig. 2A, B), in frontal view 1.4× as wide as high. Vertex and face strongly reticulate, only clypeus smooth, and meshes of reticulate sculpture around antennal toruli smaller than other parts. Vertex along occipital margin a with distinct transverse carina. POL:OOL ~ 2.8:1.0. Frontal sulcus absent. HE:MS:WM ~ 2.5:1.0:1.5. Gena curved and convex. Anterior margin of clypeus arc-shaped, slightly produced, not truncated. Antenna inserted just above level of lower margin of eyes, with 3-segmented funiculus and 2-segmented clava (Fig. 2C). Scape ~ 4.7× as long as wide, subequal to the combined length of pedicel and F1. Pedicel ~ 1.8× as long as wide, shorter than F1 (8:11). Flagellomeres decreasing in length from F1 to C2. Funiculars separated by short petioles, F1 ~ 2× as long as wide, distinctly longer than F2 (9:11). Clava longer than F1 (13:11), C1 ~ 1.2× as long as wide, C2 narrower than C1 (3:4), with terminal spine nearly 1/2 length of C2.

***Mesosoma*** (Fig. 2D, E), 1.5× as long as wide. Pronotum, mesoscutum, mesoscutellum, and metascutellum with coarse polygonal reticulation, only lateral panels of metanotum and propodeum smooth, without any trace of reticulation. Pronotum short, with weak transverse pronotal carina. Median area of the mesoscutum with two pairs of setae, and posterior margin slightly emarginate. Notauli incomplete and indicated posteriorly by a depression. Mesoscutellum 1.15× as long as wide, oval-shaped, with one pair of setae. Metascutellum very short. Propodeum long, ~ 0.42× as long as mesoscutellum, with single median carina which split and diverge anteriorly, without plica. Fore wing broad, ~ 2× as long as wide, lower surface of costal cell with a row of short setae close to SMV and extending from base to 2/3 length of the cell (Fig. 2J). Basal cell with four setae. Speculum small, and open towards base of wing. STV and PMV short, ratio of length of SMV:MV:PMV:STV ~ 5.4:12.8:1.3:1.0. Hind wing 3.4× as long as wide, apex rounded (Fig. 2K). Legs with metatibial spur slightly curved, longer than the width of apex of tibia (7:5), but not reaching apex of basal tarsomere (Fig. 2F–H).

***Metasoma*** (Fig. 2I), petiole long, at least 1.5× as long as wide. Gaster ovate, 1.5× as long as wide, and shorter than mesosoma (17:20). First gastral tergite occupying 1/3 length of gaster, with posterior margin strongly curved. Ovipositor occupying nearly the complete length of gaster, and slightly exserted beyond apex of gaster.

**Male.** Unknown.

#### Host.

Unknown.

#### Etymology.

The specific name refers to the yellow femora (*flavus* is Latin for yellow).

#### Distribution.

China (Tibet).

#### Remarks.

*Entedonflavifemur* sp. nov. is close to *E.magnificus* (Girault & Dodd, 1913) (Girault 1913). Based on the re-description of *E.magnificus* by Gumovsky et al. (2015), the two species share the following characteristics: body mainly metallic green with golden-yellow or copper reflections in some parts; scape, all femora and tibiae yellow to pale yellow; and propodeum smooth and shiny. The new species differs from *E.magnificus* in having F1 subconical, distinctly longer than F2 (vs. F1 subcircular, only slightly longer than F2 in *E.magnificus*); fore wing with an oval-shaped infuscate spot below MV (vs. hyaline in *E.magnificus*); speculum small (vs. distinctly larger in *E.magnificus*); petiole longer than wide (vs. wider than long in *E.magnificus*); and propodeum without longitudinal channel on both sides of median carina (vs. with longitudinal channel in *E.magnificus*).

### ﻿ Entedon albifemur

Taxon classificationAnimaliaHymenopteraEulophidae

Kamijo, 1988

6FFF0B66-9F2D-5698-A2B8-0B1EFD60B98A

Figs 3 , 4A–K

Entedon albifemur Kamijo, 1988: 334, ♀♂, holotype ♀, Japan, EIHU, not examined.

#### Material examined.

5♀: 3♀ [NEFU; 2 on cards, 1 on slide], China, Liaoning Province, Fushun City, Dahuofang Forestry Station, 18.VI.2012, leg. Xiang-Xiang Jin, Hui Geng and Jiang Liu, by sweep netting; 2♀ [NEFU; 1 on card, 1 on slide], China, Liaoning Province, Huludao City, Jianchang County, Bailangshan National Nature Reserve, 26.VII.2013, leg. Guo-Hao Zu, Ye Chen and Chao Zhang, by sweep netting.

#### Diagnosis.

**Figure 3.**
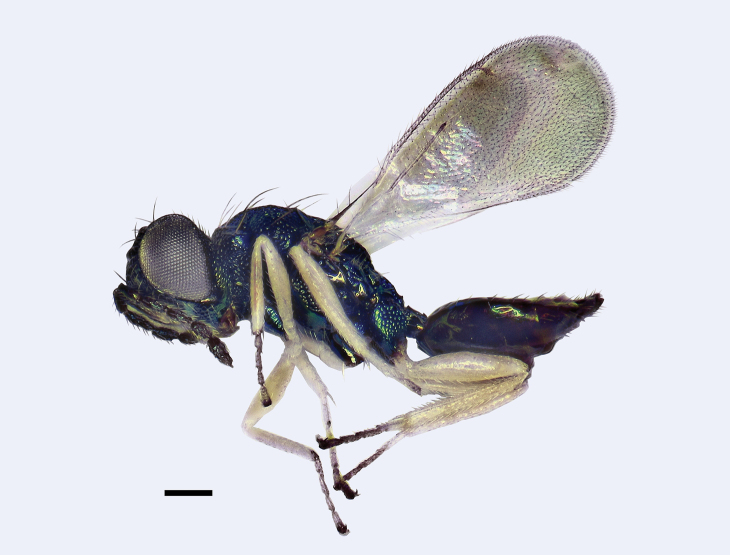
*Entedonalbifemur* Kamijo, female, habitus in lateral view. Scale bar: 200 μm.

**Figure 4.**
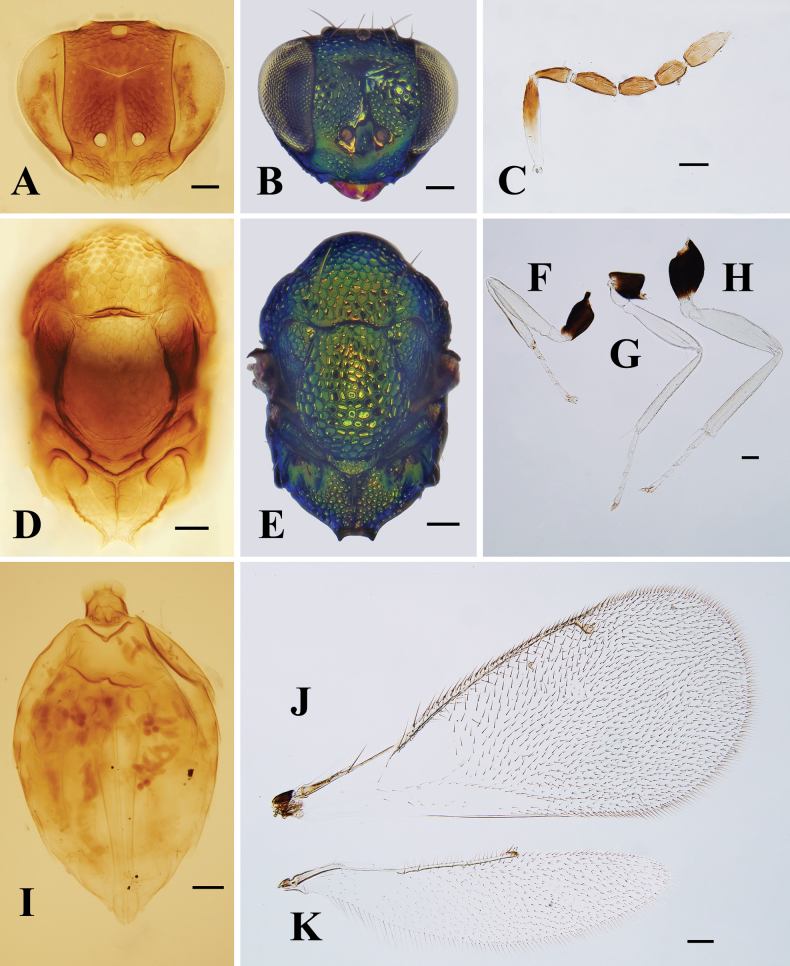
*Entedonalbifemur* Kamijo, female, on slide **A, B** head, frontal view **C** antenna **D, E** mesosoma **F–H** fore, mid and hind leg, respectively **I** metasoma **J** fore wing **K** hind wing. Scale bars: 100 μm.

**Female.** Antenna mainly dark brown, with bluish green or purple reflections, only base of scape white. All femora and tibiae white, protibia with one longitudinal brown to dark brown stripe dorsally, coxae metallic bluish green (Figs 3, 4F–H). Head strongly reticulate, with smooth transverse area above clypeus and well-developed frontal sulcus (Fig. 4A, B). Antennal scape slender, 5.2× as long as wide; pedicel ~ 2.5× as long as broad; F1 2.3–2.7× as long as wide, 1.0–1.2× as long as pedicel (Fig. 4C). Median area of propodeum with dense reticulation, but callus smooth (Fig. 4D, E). Fore wing hyaline; speculum closed; costal cell bare, with anterior margin slightly curved (Fig. 4J). Petiole quadrate or slightly wider than long. Gaster distinctly shorter than mesosoma.

**Male.** Not collected from China. According to Kamijo (1988), differs distinctly from female as follows: scape swollen in median part, 2.5× as long as wide; pedicel 2× as long as wide; funicular segments separated by distinct petioles.

#### Host.

Unknown.

#### Distribution.

China (Liaoning Province) (new record), Japan (Kamijo 1988), Far East Russia (Gumovsky 1998).

#### Remarks.

The two specimens we collected from Bailangshan National Nature Reserve have the F1 twice as long as wide, shorter than those of the type specimens, and barely longer than the F2; other characteristics are the same as in the type.

### ﻿ Entedon crassiscapus

Taxon classificationAnimaliaHymenopteraEulophidae

Erdös, 1944

A795B2BB-96E0-56EC-81B8-4A25E4747283

**Figure 5.**
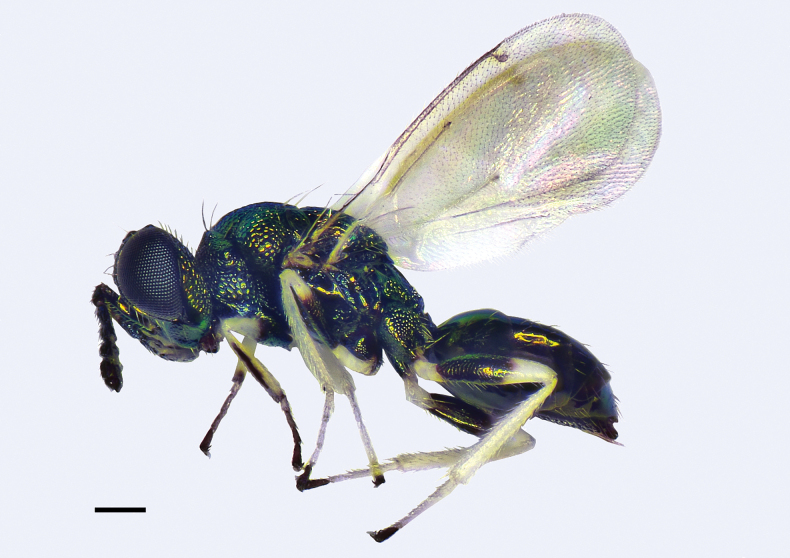
*Entedoncrassiscapus* Erdös, female, habitus in lateral view. Scale bar: 200 μm.

**Figure 6.**
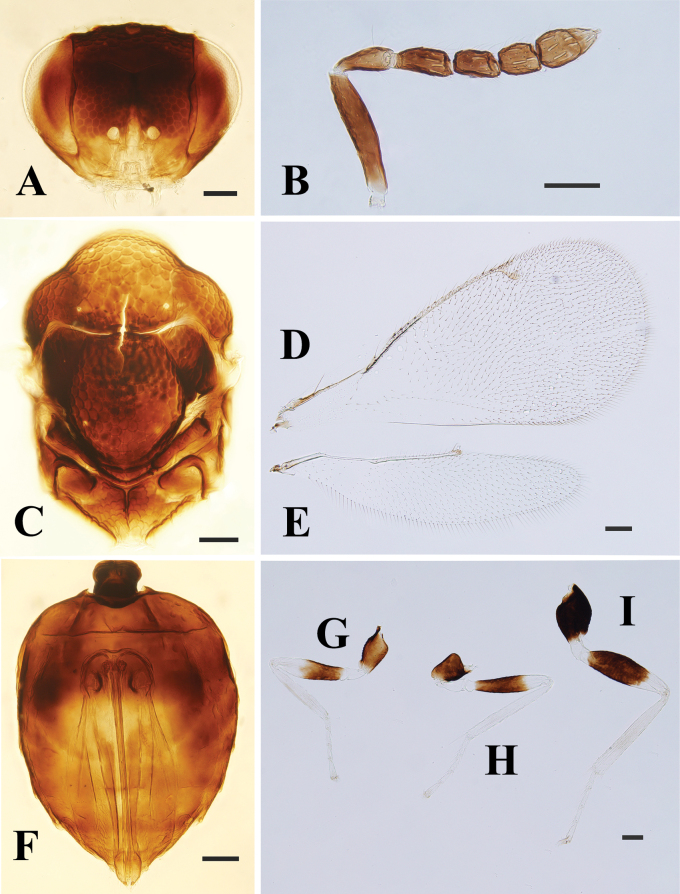
*Entedoncrassiscapus* Erdös, female, on slide **A** head, frontal view **B** antenna **C** mesosoma **D** fore wing **E** hind wing **F** metasoma **G–I** fore, mid and hind leg, respectively. Scale bars: 100 μm.

**Figure 7.**
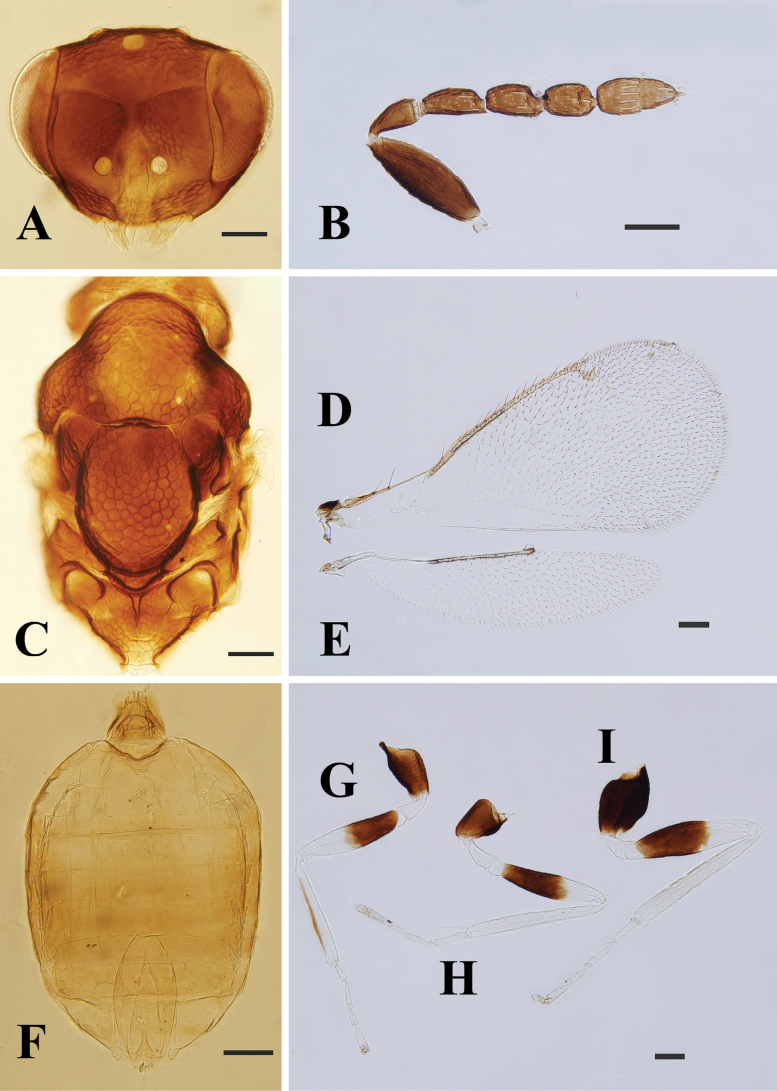
*Entedoncrassiscapus* Erdös, male, on slide **A** head, frontal view **B** antenna **C** mesosoma **D** fore wing **E** hind wing **F** metasoma **G–I** fore, mid and hind leg, respectively. Scale bars: 100 μm

Figs 5 , 6A–I , 7A–I

Entedon (Trochentedon) crassiscapus Erdös, 1944: 61, ♀, holotype ♀, Romania, HNHM, not examined.
Entedon (Dolichentedon) flavicrus Erdös, 1944: 38, ♀, lectotype ♀, Hungary, HNHM, not examined. [Synonymized by Bouček and Askew 1968: 80]

#### Material examined.

17♀ 3♂: 4♀, 1♂ [NEFU; 1♀ on card, 3♀, 1♂ on slides], China, Liaoning Province, Anshan City, Mountain Qian Shan, 25.VI.2015, leg. Hui Geng, Si-Zhu Liu, Zhi-Guang Wu and Yan Gao, by sweep netting; 1♀ [NEFU; on card], China, Liaoning Province, Anshan City, Mountain Qian Shan, 23.VI.2015, leg. Hui Geng, Si-Zhu Liu, Zhi-Guang Wu and Yan Gao, by sweep netting; 1♀, 1♂ [NEFU; on cards], China, Liaoning Province, Anshan City, Mountain Qian Shan, 22.VI.2015, leg. Hui Geng, Si-Zhu Liu, Zhi-Guang Wu and Yan Gao, by sweep netting; 1♀, 1♂ [NEFU; on cards], China, Liaoning Province, Anshan City, Mountain Qian Shan, 16.IX.2015, leg. Hui Geng, Ye Chen and Xin-Yu Zhang, by sweep netting; 3♀ [NEFU; on cards], China, Liaoning Province, Fushun City, Dahuofang Forestry Station, 18.VI.2012, leg. Xiang-Xiang Jin, Hui Geng and Jiang Liu, by sweep netting; 2♀ [NEFU; on cards], China, Liaoning Province, Fushun City, Dahuofang Forestry Station, 19.VI.2012, leg. Xiang-Xiang Jin, Hui Geng and Jiang Liu, by sweep netting; 1♀ [NEFU; on card], China, Liaoning Province, Fushun City, Qingyuan County, Shimengou, 10.VI.2012, leg. Xiang-Xiang Jin, Hui Geng and Jiang Liu, by sweep netting; 2♀ [NEFU; on cards], China, Liaoning Province, Huludao City, Jianchang County, Bailangshan National Nature Reserve, 4.VII.2012, leg. Si-Zhu Liu and Jiang Liu, by sweep netting; 1♀ [NEFU; on card], China, Heilongjiang Province, Shangzhi City, Maoershan, leg. Ye Chen and Chao Zhang, by sweep netting; 1♀ [NEFU; on card], CHINA, Inner Mongolia, Yakeshi City, Fenghuang Villa, leg. Yue Qin and Yuan-Yuan Jin, by yellow-pan trapping.

#### Diagnosis.

**Female.** Antennal scape dark brown to black, with bluish green reflections. All femora dark brown to black with metallic bluish green reflections in proximal 1/2 to 2/3 of its length, the remainder is white; all trochanters and tibiae white, only protibiae with a dark brown longitudinal stripe (Figs 5, 6G–I). Head strongly reticulate; frontal sulcus present and nearly complete; gena straight (Fig. 6A). Antenna with F1 almost as long as pedicel, 1.7× as long as broad, slightly longer than F2; the latter 1.5× as long as broad; F3 quadrate or slightly transverse (Fig. 6B). Fore wing hyaline, costal cell bare, speculum closed (Fig. 6D). Scutellum only slightly longer than broad. Propodeum densely reticulated (Fig. 6C). Petiole slightly wider than long. Gaster slightly shorter than mesosoma.

**Male.** Clearly differentiated from female as follows: scape swollen in median part, 2.7× as long as wide (Fig. 7B); F3 distinctly longer than wide; propodeum longer than in female.

#### Host.

Unknown from China. According to Bouček and Askew (1968), *Entedoncrassiscapus* primarily parasitize *Mordellistenaparvula* and *M.weisei* (Coleoptera, Mordellidae).

#### Distribution.

China (Heilongjiang, Liaoning, and Inner Mongolia) (new record), Hungary (Erdös 1944), Germany, Romania, Italy (Bouček and Askew 1968), Azerbaijan, Croatia, Montenegro, Spain (Bouček 1977), Czech Republic, Slovakia (Kalina 1989), Sweden (Hansson 1991), Ukraine, Far East Russia, Japan, Korea (Gumovsky 1998), Bulgaria (Gumovsky and Boyadzhiev 2003).

### ﻿ Entedon nomizonis

Taxon classificationAnimaliaHymenopteraEulophidae

Kamijo, 1988

A6688B2E-76BA-5158-AC1C-45EC51995F07

Fig. 8A–I

Entedon nomizonis Kamijo, 1988: 331, ♀♂, holotype ♀, Japan, EIHU, not examined.

#### Material examined.

1♀ [NEFU; on slide], China, Liaoning Province, Huludao City, Jianchang County, Bailangshan National Nature Reserve, 4.VII.2012, leg. Si-Zhu Liu and Jiang Liu, by sweep netting.

#### Diagnosis.

**Figure 8.**
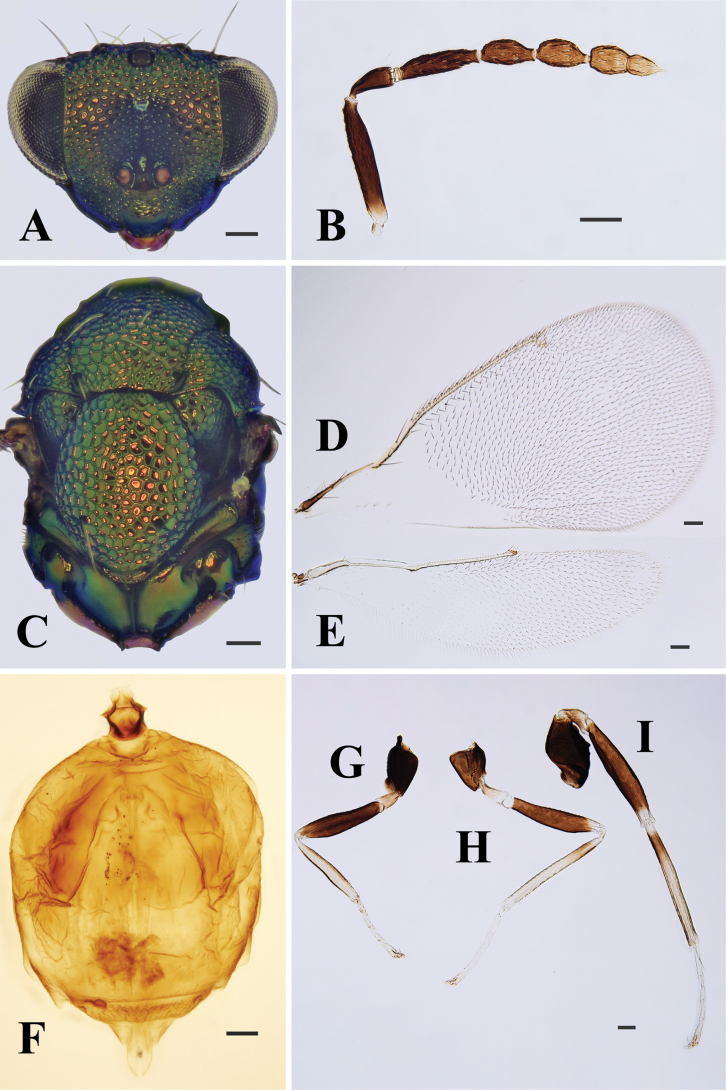
Entedonnomizonis Kamijo, female, on slide **A** head, frontal view **B** antenna **C** mesosoma **D** fore wing **E** hind wing **F** metasoma **G–I** fore, mid and hind leg, respectively. Scale bars: 100 μm.

**Female.** Scape dark metallic blue. All coxae and femora metallic bluish green, only apex of all femora pale yellow or white; trochanters brown to black; tibiae mainly pale yellow to white, protibiae dorsally and ventrally dark brown, mesotibiae and metatibiae with single dark brown band basally. Head strongly reticulate, only clypeus smooth; frontal sulcus absent; gena almost straight; clypeus convex, with anterior margin produced and distinctly curved (Fig. 8A). Scape slender, 5.5× as long as wide; pedicel slightly > 2× as long as wide; F1 3.0–3.8× as long as wide (including measurements from the original description, the same below), 1.5–2.0× as long as pedicel, distinctly longer than F2; clava nearly as long as F1 (Fig. 8B). Propodeum smooth and shiny (Fig. 8C). Fore wing hyaline, costal cell bare, with anterior margin distinctly curved; speculum completely open below (Fig. 8D). Metatibial spur long and slightly curved, nearly reaching apex of basal tarsomere (Fig. 8I). Petiole longer than wide. Gaster shorter and wider than mesosoma.

**Male.** Not collected from China. According to Kamijo (1988), differs from female as follows: scape swollen in median part, 2.4–2.7× as long as wide; all five flagellar segments separated by distinct petioles, filiform, hardly stouter than pedicel; F1 nearly 6× as long as wide, narrowed medially; petiole 1.7× as long as wide, and distinctly longer than propodeum.

#### Host.

Unknown from China. According to Kamijo (1988), *Entedonnomizonis* primarily parasitizes *Rhynchaenusjaponicus*, *R.sanguinipes*, and *R.takabayashii* (Coleoptera, Curculionidae).

#### Distribution.

China (Liaoning Province) (new record) and Japan (Kamijo 1988).

## Supplementary Material

XML Treatment for Entedon flavifemur

XML Treatment for Entedon albifemur

XML Treatment for Entedon crassiscapus

XML Treatment for Entedon nomizonis

## ﻿Additional information

### Author ORCIDs

Ming-Rui Li https://orcid.org/0000-0002-9143-1548

### Data availability

All of the data that support the findings of this study are available in the main text.
